# Factors influencing catastrophic health expenditure of households with people with diabetes in Northwest China-an example from Gansu Province

**DOI:** 10.1186/s12913-023-09411-w

**Published:** 2023-04-25

**Authors:** Ying Dang, Yinan Yang, Aimin Yang, Shuting Cao, Jia Zhang, Xiao Wang, Jie Lu, Xiaobin Hu

**Affiliations:** 1 Department of Epidemiology and Statistics, School of Public Health, Lanzhou University, Lanzhou, Gansu, China; 2 Department of Pediatric Cardiology, Lanzhou University Second Hospital, Lanzhou, Gansu, China; 3 Department of Medicine & Therapeutics, The Chinese University of Hong Kong, Hong Kong SAR, China; 4 Health Statistics Information Center of Gansu Province, Lanzhou, Gansu Province, China

**Keywords:** Households, Diabetes, Health economics, Catastrophic health expenditure, Risk factors, Equity analysis, Decision tree

## Abstract

**Background:**

Diabetes is a chronic non-communicable disease that causes a substantial economic burden on diabetic suffers and their households. The aim of this study was to explore the prevalence, equity, and determinants of catastrophic health expenditure (CHE) among households with people with diabetes in Northwest China.

**Methods:**

A total of 3,000 households were included in the 6th Health services survey in Gansu Province, China of which 270 households with people with diabetes. The equity of CHE was evaluated by concentration curve and concentration index (CI). We adopted the Pareto chart to analyze the main economic intervals of the occurrence of CHE. Finally, we combined the decision tree and logistic model and analyzed the determinants of the occurrence of CHE.

**Results:**

The incidence of CHE at 15%, 25% and 40% were 75.19%, 58.89% and 35.19%, respectively. CHE tended to occur in households with a lower economic level, with the phenomenon being more pronounced at Z = 40%. The Pareto chart showed that households in the group with an annual per capita income of 0–740 USD (0–5,000 Chinese Yuan) were most likely to experience CHE. Both decision tree and logistic models suggested that economic level, comorbidities, and small household size were potential risk factors. In addition, the decision tree model also suggested the interaction between the influencing factor of health checks in the past 12 months and the number of chronic diseases.

**Conclusions:**

In summary, Households with people with diabetes were more likely to incur CHE. It is essential to focus on low- and middle-income households with people with diabetes, strengthen the management of patients with diabetes, and provide timely health interventions to reduce the occurrence of chronic comorbidity and the risk of CHE in households.

## Background

Globally, 10.6% of the world's population were affected by diabetes, the majority having type 2 diabetes (T2D), with major healthcare and socioeconomic implications [1]. The number of diagnosed and undiagnosed people with diabetes in China ranks first in the world, with the number of diagnosed diabetics increasing by 56% from 2011 to 2021 [2]. Over 3 in 4 adults with diabetes live in low- and middle-income countries. Health expenditures related to diabetes have increased 316% to $966 billion over the past 15 years around the world, and China ranked at second globally in diabetes-related health expenditures with $165.3 billion [1].

Catastrophic health expenditure (CHE) means that a household's health care expenses account for a certain percentage of the household's disposable income [3]. The incidence and gap of CHE are two commonly used indicators to reflect the breadth and depth of health expenditure of a specific population. In 1998, China officially established a social medical insurance system. A study from Ghana showed that the National Health Insurance Scheme (NHIS) could provide financial protection for households and reduce their likelihood of CHE [4]. The cumulative incidence of CHE was 10.3% per month in Nepal, and households living in the West are more likely to occur with CHE [5]. Over two decades of development of Chinese social medical insurance system since 1999, the proportion of costs reimbursed has been steadily increasing based on the basic realization of full coverage of medical costs for the residents in China [6]. Health insurance systems can provide households with some financial protection and reduce their risks of incurring CHE, with different approaches offering varying degrees of protection. In recent years, China experienced a 0.70-fold change in the incidence of CHE, especially for poorer groups, between 2010 (12.57%) and 2016 (8.94%) [7]. A comparative analysis using 2013 survey data showed that the incidence of CHE in areas implementing the Integration of Urban and Rural Medical Insurance Scheme IURMIS (13.68%) was lower than that in areas that did not implement the IURMIS (13.87%) [8]. As Chinese medical insurance system entered a post-reform era, the incidence of CHE dropped from 19.37% to 15.11%, according to a study from 2010 to 2016 [8]. A Chinese longitudinal study showed that the incidence of CHE was related to the treatment taken by cancer patients, with patients receiving chemotherapy (OR = 2.53) and surgery (OR = 2.15) having a significantly increased risk of CHE in their households [9]. Another cross-sectional study in China showed that for each additional chronic disease, the probability of CHE increased by 39% [10]. Several studies have shown that household economic level was one of the key factors influencing the occurrence of CHE in households, with an increased level of household income decreasing the incidence of CHE [11, 12]. In addition, Medical insurance, the gap between urban and rural areas, the elderly population and the utilization of health services significantly affected the fairness of CHE [8].

Northwest of China, an economically underdeveloped region, has an 11.16% prevalence of diabetes﻿ [13]. Gansu Province, located in northwest of China and the upper reaches of the Yellow River, is an economically underdeveloped region of China, along with a relatively dispersed population distribution. Limited medical resources, low health insurance coverage, and inadequate public health education are important barriers to effective management of diabetes in the region. Several studies have focused on the determinants of CHE and economic burden of diabetes, but limited data is available for diabetes in rural area of northwest China [14, 15]. In the present study, we investigated the prevalence of CHE in households with people with diabetes in Northwest China, and explored the potential risk factors associated with CHE.

## Methods

### Data source and participants

**Fig. 1:**
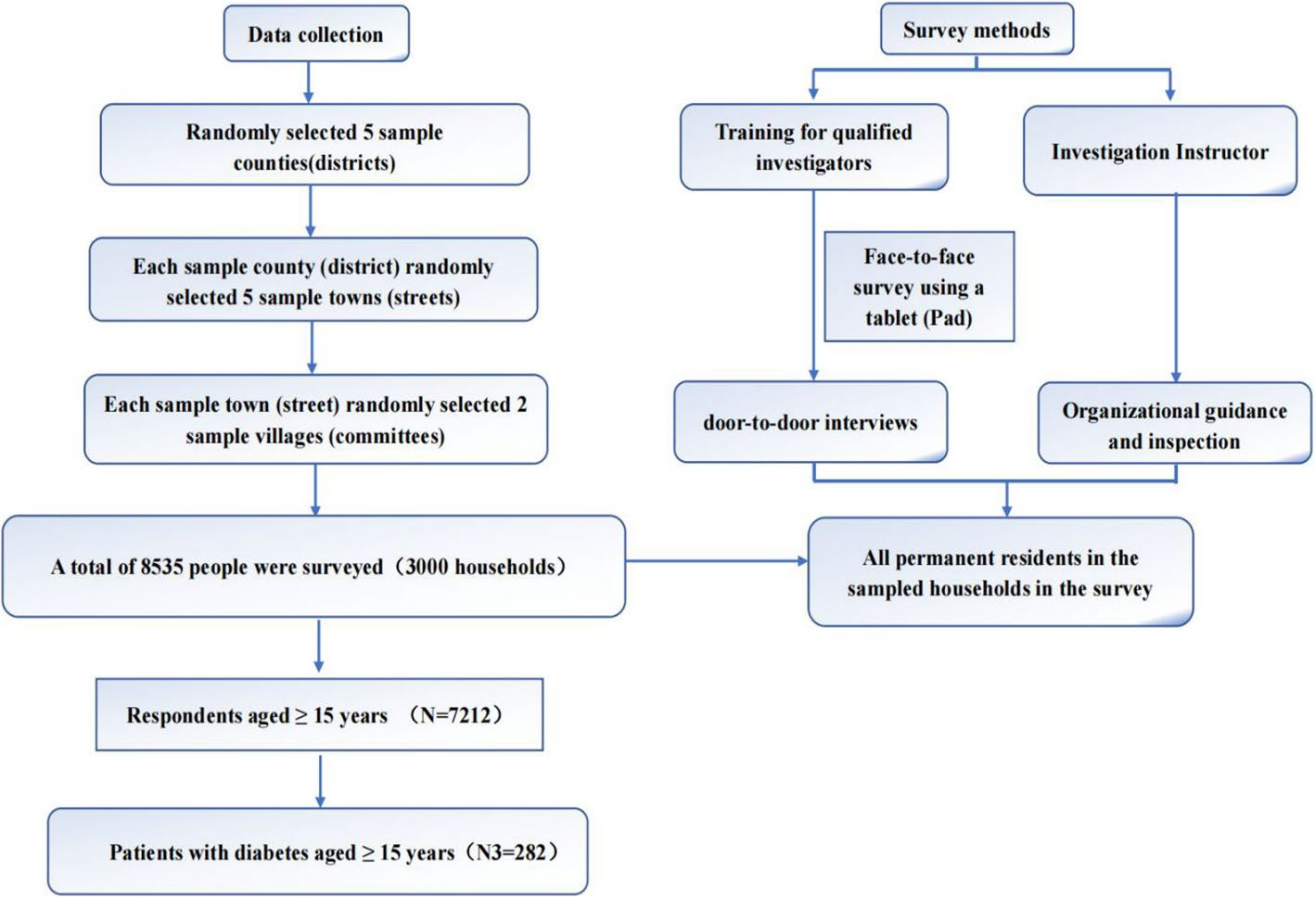
Data collecting process

This study was carried out in Gansu province, China. We used data from the Chinese Sixth National Health and Services Survey (NHSS) in Gansu province from August 2018 to October 2018, led by the Health Commission of Gansu Province [16]. This survey adopted multi-stage random sampling of the whole group, following the principle of economic validity while ensuring the representativeness of the sample and assessing the whole population through the sample. First, five districts/counties were selected (Yuzhong, Jingtai, Lintan, Maiji, Ganzhou). Second, all towns (streets) in each selected district (counties) were divided into five levels, and one town or street is randomly selected at each level. Third, two villages (residential committees) were randomly selected from each selected town or street. Figure 1 shows the flowchart of the selections (See Fig. 1).

People with diabetes was measured by the question, "Have you been diagnosed with diabetes?" If the answer was "yes", diabetes was coded as "1" and if the answer was "no", diabetes was coded as "0", but excluded those who had a new episode within the two weeks prior to the survey. Following the rigorous data screening process, 282 people with diabetes were selected as the subjects of the study, 12 of whom were from the same households.

### Data collection

The NHSS has been conducted nationwide every five years since 1993, aiming to reflect the level of medical security, health service system construction, residents' health status, residents' demand for health services, and residents' demand for medical services. The Investigators used tablet computers (PADs) to go door-to-door to residents' homes to conduct face-to-face surveys from August to October, 2018. To ensure quality, all of the completed questionnaires were carefully checked by supervisors after the interview each day.

### Variables selection

Based on the Anderson Health Services Model, a total of 20 variables were selected by combining the relevant variables from the health services survey questionnaire [17]**.** The variables were divided into 4 major categories for the analysis of CHE influencing factors. This can provide ideas for policy makers to develop appropriate policies for diabetic patients in the northwest of China, so that they may better provide certain economic protection and improve the health of the whole population and the quality of health services.

### CHE assessment/threshold

Outcomes of interest in this study included incidence and gap of CHE. The incidence of CHE can indicate the breadth of households affected by CHE among all households with people with diabetes. In Eq. (1), the indicator Ei is used to show whether CHE occurred, and Z is the threshold for the occurrence of CHE, occurring as 1 or not occurring as 0. In Eq. (2), HE means health expenditure, HDI shows disposable household income.

1 $${E}_{i}=\left\{\begin{array}{c}0, CH{E}_{i}<Z\\ 1, CH{E}_{i}\ge Z\end{array}\right.$$

2 $$CH{E}_{i}=\frac{H{E}_{i}}{HD{I}_{i}}$$

Both AGCHE (Average Gap in Catastrophic Health Care Expenditures) and RGCHE (Relative Gap in Catastrophic Health Care Expenditures) are indicators used to measure the financial burden of household health care expenditures. AGCHE reflects the intensity of the economic gap for households who incurred CHE in all of those. It can be used to assess the average economic burden of catastrophic medical expenditures for different income groups. Higher AGCHE values indicate a greater economic burden of CHE, which may indicate the need for policy interventions to reduce this burden. In Eq. (3), N represents the number of surveyed households. RGCHE reflects the intensity of the economic gap between households who incurred CHE on those who incurred CHE. Higher RGCHE values indicate higher economic inequality in health care spending, which may indicate the need for policy interventions to promote equity in health care delivery. In Eq. (4), n denotes the number of households that incurred CHE [18].

3 $$AGCHE=\frac{1}{N}\sum\nolimits_{i=1}^{N}{E}_{i}\left(CH{E}_{i}-Z\right)$$

4 $$RGCHE=\frac{1}{n}\sum\nolimits_{i=1}^{N}{E}_{i}\left({\mathrm{CHE}}_{\mathrm{i}}-Z\right)$$

The distribution of CHE related to household economic level among households with people with diabetes is uneven [19]. Wagstaff introduced Concentration Index (CI) in 1991, a quantitative standard with a value between -1 and 1 [20]. When the CI is less than 0, it indicates that CHE is more likely to occur in households with low economic levels, and when CI is greater than 0, it means the opposite. The calculation of CI shows in Eq. (5). Where h represents the health index of the population, Ri represents the fractional rank of the sample I related to income, variance represents the covariance of the relative rank of calculated variables, and n represents the sample size.

5 $$CI\left(\mathrm{h}|\mathrm{y}\right)=\frac{2cov\left({\mathrm{h}}_{\mathrm{i}},{R}_{i}\right)}{\overline{\mathrm{h}} }=\frac{1}{n}\sum\nolimits_{i=1}^{n}\left[\frac{{\mathrm{h}}_{i}}{\overline{\mathrm{h}} }\left(2{R}_{i}-1\right)\right]$$

Because this study chooses indicators of CHE as two-category variables, the values have clear upper and lower limits, which belong to Bounded variables, so this study used the modified formula proposed by Wagstaff (2005) to calculate CI [21]. In Eq. (6), h_max_ and h_min_ represent the maximum and minimum values of h index in the sample, respectively.

6 $$\mathbf{W}\mathbf{a}\mathbf{g}\mathbf{s}\mathbf{t}\mathbf{a}\mathbf{f}\mathbf{f}.\mathbf{C}\mathbf{I}\left({\varvec{h}}|{\varvec{y}}\right)=\frac{1}{{\varvec{n}}}\sum_{{\varvec{i}}=1}^{{\varvec{n}}}\left[\frac{\left({{\varvec{h}}}^{{\varvec{m}}{\varvec{a}}{\varvec{x}}}-{{\varvec{h}}}^{{\varvec{m}}{\varvec{i}}{\varvec{n}}}\right){{\varvec{h}}}_{{\varvec{i}}}}{\left({{\varvec{h}}}^{{\varvec{m}}{\varvec{a}}{\varvec{x}}}-\overline{{\varvec{h}} }\right)\left(\overline{{\varvec{h}} }-{{\varvec{h}}}^{{\varvec{m}}{\varvec{i}}{\varvec{n}}}\right)}\left(2{{\varvec{R}}}_{{\varvec{i}}}-1\right)\right]$$

In this study, the dependent variable was whether CHE occurred. Independent variables could be subcategorized into 4 types: demographic and social characteristics, family characteristics, health and illness characteristics, health services utilization characteristics. The criteria for judging whether CHE has occurred varies from country to country and are generally determined by family living standards. In order to maintain comparability of the studies, descriptive analyses were conducted under three thresholds (15%, 25%, and 40%), with 40% defining criteria for the effect factors in this study.

### Statistic analyzing

We calculated the incidence of CHE, AGCHE, and RGCHE by different economic degrees. We explored the incidence of CHE in different income groups by using Pareto charts to identify what kind of households were most likely to incur CHE. It has been shown that the decision tree model can be applied to screen influencing factors, and in some cases, compared to logistic regression models. Decision tree models can reveal the influencing factors more comprehensively and also visualize the correlation between them [22]. In this study, decision trees and logistic regression were used to analyze the determinants of catastrophic health expenditure. The classification and regression tree (CART) algorithm was used to generate a decision tree model, and the following parameters were chosen: the Gini coefficient was used as the basis for classification; the maximum depth of the decision tree was set to 5, and the minimum sample required to split the internal nodes was 20. Both of the models were evaluated by sensitivity under the ROC curve and area under the curve (AUC). The value of the AUC varies from 0 to 1, and the closer the value is to 1, the higher the diagnostic accuracy of the method is represented. As household income and expenditure in this survey were surveyed in Chinese Yuan (CNY), to make the results more comparable to other published studies, we converted to US$ using the average exchange rate in 2018 (1USD = 6.75CNY).

### Ethic statement

The Ethics Committee of the School of Public Health, Lanzhou University reviewed and approved the study protocols and instruments. Written informed consent was obtained from all of the participants before the interview.

## Results

### The status of CHE under different thresholds

The incidence of CHE, AGCHE, and RGCHE showed a decreasing trend as the defining criteria (subsequently denoted by Z) increased (See Table 1). The decline between Z = 15% and Z = 25% (16.30 percentage points) was lower than the decline between Z = 25% and Z = 40% (23.70 percentage points).

**Table 1:** CHE occurrence among households with diabetic patients

Z	Number of occurrences (households)	Incidence of CHE (%)	AGCHE(%)	RGCHE(%)
15%	203	75.19	18.62	24.76
25%	158	58.89	11.96	20.43
40%	95	35.19	5.09	14.47

### Distribution of CHE by income quintile

We divided all households into five groups based on annual per capita household income, from the poorest (1st quintile) to the wealthiest (5th quintile) households. The overall trend of the incidence of CHE declined as the economic level rose under the same defined criteria. The AGCHE did not show a clear changing pattern across economic levels. RGCHE was higher in the low-income group than in the other groups (except for Z = 40%). The defined criteria showed a negative correlation with each group's incidence of CHE, AGCHE, and RGCHE at a given economic level (See Table 2).

**Table 2:** Percentages of occurrence of catastrophic in households with diabetes at different economic levels

Z	Household economic status^a^	Incidence of CHE(%)	AGCHE(%)	RGCHE(%)
15%	Quintile 1	84.21	25.65	30.46
	Quintile 2	82.61	16.75	20.27
	Quintile 3	62.50	13.38	21.41
	Quintile 4	80.39	23.04	28.66
	Quintile 5	69.23	14.66	21.17
30%	Quintile 1	84.21	25.65	30.46
	Quintile 2	70.18	18.14	25.85
	Quintile 3	56.52	9.78	17.31
	Quintile 4	48.44	7.89	16.29
	Quintile 5	66.67	15.55	23.32
40%	Quintile 1	56.14	8.71	15.51
	Quintile 2	28.26	3.64	12.88
	Quintile 3	23.44	2.94	12.56
	Quintile 4	43.14	7.44	17.25
	Quintile 5	25.00	2.75	11.01

^a^Quintile 1 was the poorest and quintile 5 was the wealthiest

### Analysis of equity

**Fig. 2:**
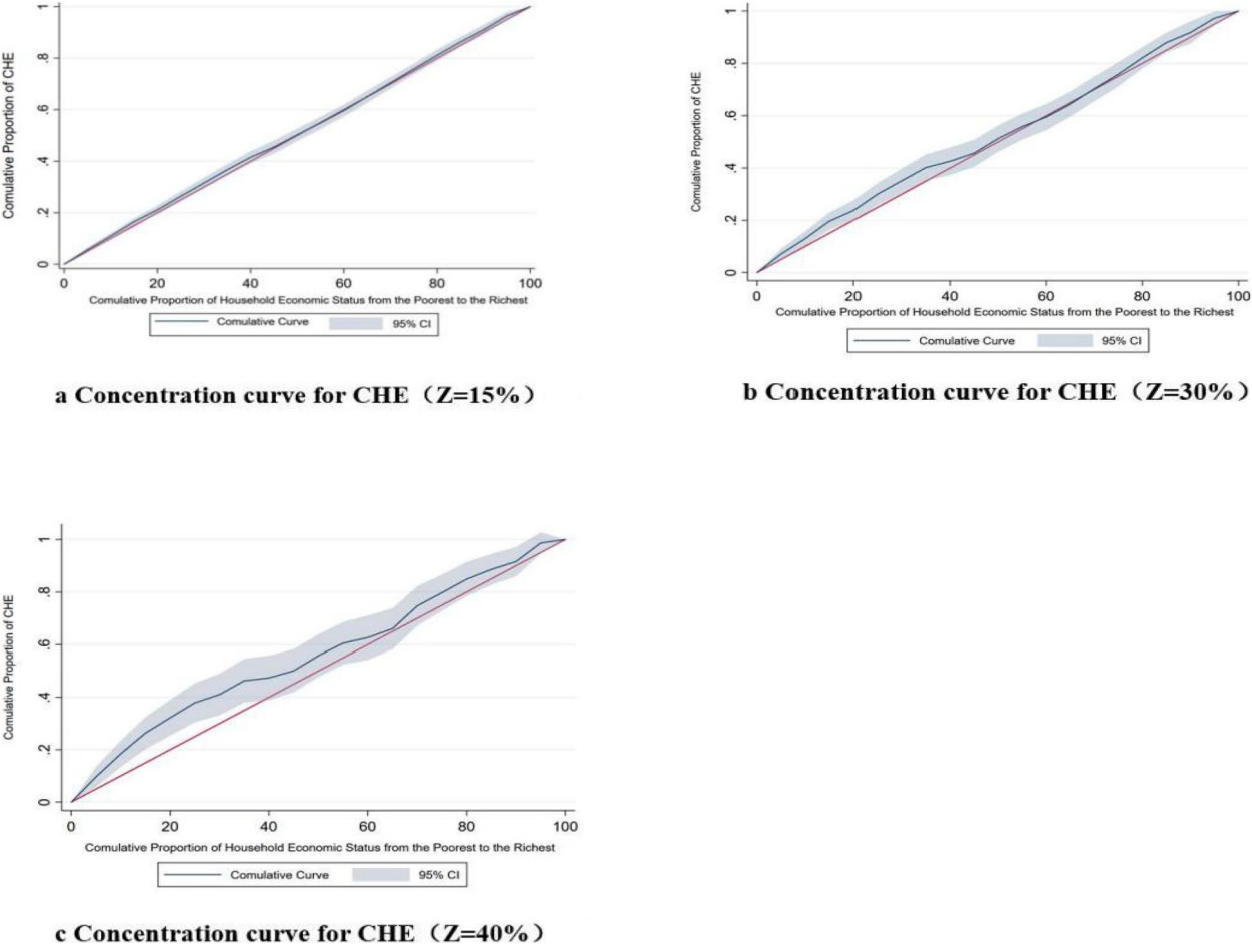
Concentration curve for CHE in different criteria

By analyzing the CI with different threshold (See Table 3), we found that when the thresholds were 15%, 25%, and 40%, CIs were all negative (-0.1234, -0.1044, and -0.1885), indicating that low-income households easily experienced CHE. Comparing the CI of households with different living environments, the above phenomenon was more pronounced in rural areas. In particular, when Z was 40% (CI = -0.3475), low-income households living in rural areas were more likely to be stuck in CHE (See Table 3). The Fig. 2 represented the concentration curves under different criteria. We could see that no matter where households lived in rural or urban areas, the cumulative curve was above the equity line, suggesting that CHE occurred more in low-income households. The tendency was more noticeable when Z = 40% (See Fig. 2c).

**Table 3:** Equity of CHE in households with people with diabetes

Z	Urban		Rural		Total	
	**CI**	**Std**	**CI**	**Std**	**CI**	**Std**
15%	-0.7344**	0.2329	-0.1754	0.1126	-0.1234	0.1022
25%	-0.4507	0.1424	-0.1674*	0.8222	-0.1044	0.0713
40%	-0.0774	0.1422	-0.3475***	0.0833	-0.1885*	0.0729

^*^meant *P < *0.05; **meant *P < *0.01; ***meant *P < *0.001

### Pareto char of CHE

**Fig. 3:**
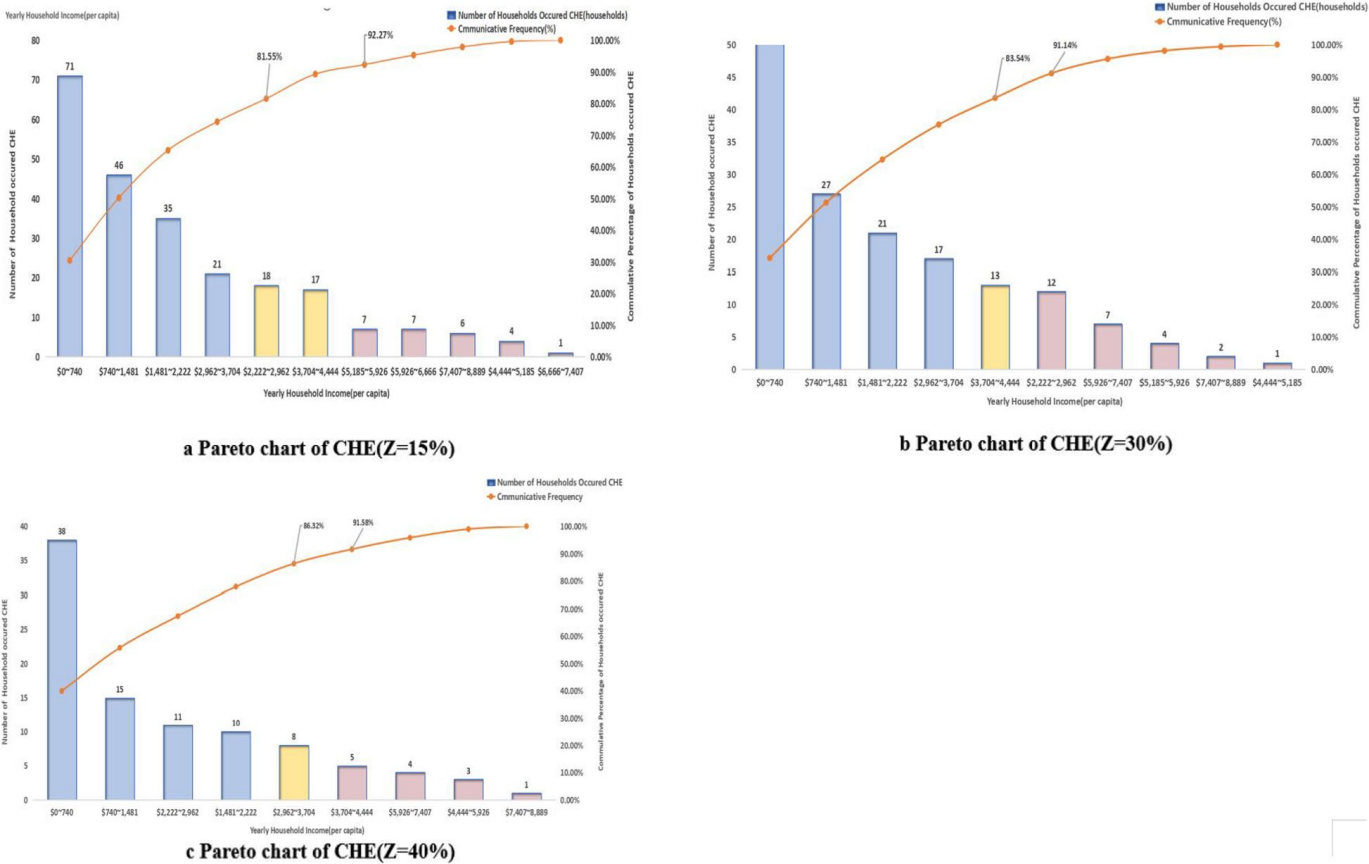
Pareto chart of CHE

According to the different annual per capita income groups corresponding to the cumulative percentage of incidence of CHE, there were three intervals: the cumulative percentage of occurrence within 80% was the A interval, which was the main economic interval in which CHE occurred and was also the main factor interval we need to pay attention to; 80% to 90% was the B range, which is the secondary economic range; the cumulative percentage of occurrence of 90% or more was the C range, which is the least important factor. Figure 3 indicated that households with annual per capita income groups of 0-740USD (0–5,000 CNY) were most likely to have CHE. The main groups where CHE occurred under the three defined criteria were under 2,962 USD (19,994CNY), under 4,444 USD (30,000 CNY), and under 3,704 USD (25,000 CNY) or less (See Fig. 3).

### Single determinants of catastrophic health expenditure

Regarding demographic and sociological factors, we could see that as age increased, the incidence of CHE became higher. In terms of household elements, the number of household members, whether the household has older people over 65 years old, annual per capita income, and whether it was a low-income household were the main factors affecting the occurrence of CHE. In terms of health and morbidity factors, the results of the univariate analysis of CHE were significant for physical pain, the number of types of chronic diseases, whether health checks were conducted in the last 12 months, and self-rated health status. Concerning health service utilization, the incidence of CHE was higher for diabetic patients with inpatient service utilization (46.38%) than for diabetic patients without hospitalization within one year (31.34%). (See Table 4).

**Table 4:** Analysis of single determinants influencing the occurrence of CHE

Variables	Z = 15%			Z = 25%			Z = 40%		
	**Incidence of CHE (%)**	**χ**^**2**^	**P**	**Incidence of CHE (%)**	**χ**^**2**^	**P**	**Incidence of CHE (%)**	**χ**^**2**^	**P**
**Demographic characteristics**									
Gender									
Male	76.98	0.526	0.468	58.99	0.027	0.871	34.53	0.054	0.817
Female	73.28			58.02			35.88		
Ethnicity									
Han	75.30	7.300	0.069	58.17	0.181^、^	0.670	35.86	0.705	0.401
Minority	73.68			63.01			26.32		
Age group									
15-	41.67	0.797	0.671	25.00	7.480	0.024	16.67	11.156	0.004
45-	74.81			56.30			27.41		
65	78.86			64.23			45.53		
Education level									
Illiteracy	83.56	1.069	0.785	63.01	8.014	0.046	41.10	6.601	0.086
Elementary school	77.22			65.82			40.51		
Middle school	70.00			57.14			32.86		
High school and above	66.67			41.67			20.83		
Marital status									
Married	76.79	0.638	0.425	60.27	1.658	0.198	35.71	0.161	0.688
Other	67.39			50.00			32.61		
Urban	80.00	2.105	0.147	60.00	0.085	0.77	40.00	0.961	0.327
Rural	73.50			58.00			33.50		
Household size									
1	78.57	1.590	0.451	67.86	16.596	< 0.001	46.43	12.767	0.002
2	85.00			70.00			44.17		
3 or more	64.75			45.08					
One or more elderly^a^									
Yes	73.43	0.847	0.357	55.24	1.342	0.247	44.09	8.346	0.004
No	77.17			62.20			27.27		
Health care insurance									
UEBMI^b^	76.47	0.168	0.920	60.29	2.139	0.343	37.25	3.966	0.138
Other	73.33			55.00			31.67		
None	50.00						0.0		
**Household economic characteristics**									
Household economic status^c^									
Quintile 1	84.21	9.080	0.059	70.18	8.272	0.082	56.14	19.595	< 0.001
Quintile 2	82.61			56.52			28.26		
Quintile 3	62.50			48.44			23.44		
Quintile 4	80.39			66.67			43.14		
Quintile 5	69.23			51.92			25.00		
Poverty-stricken household									
Yes	85.00	0.799	0.371	70.00	1.173	0.279	70.00	0.429	0.512
No	74.40			57.60			32.40		
Destitute Household characteristics									
Yes	87.50	0.799	0.371	75.00	1.903	0.168	62.50	0.878	0.349
No	74.41			57.48			33.46		
**Health and disease characteristics**									
Physical pain									
No problems	68.75	3.340	0.068	53.75	3.679	0.055	29.38	5.813	0.016
Medium or serious problems	84.55			65.45			43.64		
Anxiety or depression									
No problems	73.85	0.154	0.695	57.34	0.648	0.421	33.49	1.433	0.231
Medium or serious problems	80.77			63.46			42.31		
Number of chronic diseases^d^									
1	65.31	7.769	0.021	50.00	5.074	0.079	24.49	10.202	0.006
2	79.17			61.67			37.50		
3 or more	84.62			67.31			50.00		
Health Record									
Yes	75.58	3.826	0.148	58.53	2.462	0.292	34.10	1.001	0.606
No, but understand	85.71			85.71			28.57		
No, but do not understand	71.74			54.35			41.30		
Household doctor									
Yes	75.25	1.116	0.572	58.42	0.063	0.969	33.66	2.124	0.346
No, but understand	82.61			60.87			30.43		
No, but do not understand	71.11			57.78			44.44		
Health check in the past 12 months									
Yes	77.30	0.394	0.530	60.54	0.990	0.32	39.46	4.708	0.030
No	70.59			54.12			25.88		
Self-assessment of health status^e^									
Quintile 1	85.71	6.406	0.171	57.14	20.028	< 0.001	57.14	18.244	0.001
Quintile 2	94.44			83.33			50.00		
Quintile 3	91.15			69.91			46.02		
Quintile 4	81.36			44.92			22.03		
Quintile 5	78.57			50.00			28.57		
**Health services utilization**									
Biweekly Clinic	68.97	6.449	0.011	54.31	1.484	0.223	31.03	1.536	0.215
Yes	79.87			61.69			38.31		
No									
Hospitalization episode									
Yes	84.06	0.993	0.319	68.12	3.517	0.061	46.38	5.091	0.024
No		72.14			55.22			31.34	

^a^referred to the household with one or more persons aged 65 or older

^b^referred to Urban Employees Basic Medical Insurance

^c^Quintile 1 was the poorest and Quintile 5 was the wealthiest

^d^1 meant patients with only diabetes; 2 meant diabetics suffer from another chronic disease; 3 or more meant diabetic patients with two or more chronic diseases

^e^Quintile 1 was the worst and Quintile 5 was the best

### The importance of prediction variables in the cart decision tree model

**Fig. 4:**
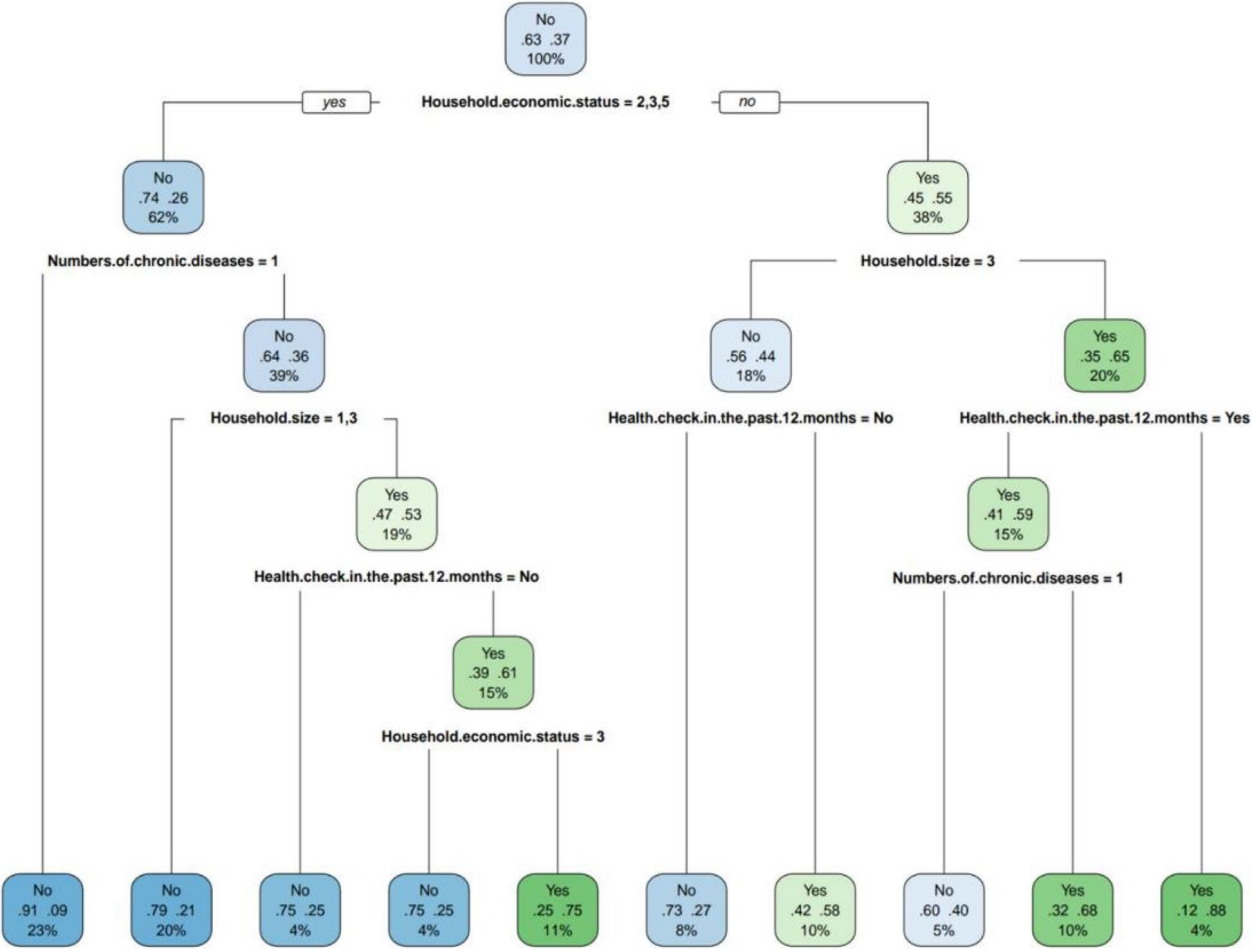
Cart decision model using Cart method

Figure 4 showed that of all the variables, decision tree analysis identified 4 important variables in the evaluation that influenced the occurrence of CHE in households with people with diabetes. No health services variables in the decision tree were founded in the decision tree. We could see that the most important predictor, which formed the root node, was household economic status. The other predictors were the number of chronic diseases, household size, and health checks in the past 12 months. The result also showed that suffering from only 1 chronic disease and physical examination in the last 12 months were protective factors for the occurrence of CHE, regardless of economic status. The incidence of CHE for households with economic status quintile 1 or 4 was 63%. The CART model also showed an interaction between household economic status and household size, with a household economic status of quintile 1 or 4 and a decrease in incidence of CHE from 55 to 44% for households with a household size of 3. There was an interaction between the presence or absence of health checks in the last 12 months and the number of chronic diseases in households with economic status of 1 or 4 and a household size of 1 or 2. Households with a household economic status of quintile 1 or 4, a household size of 1 or 2 persons, and no health check-up in the last 12 months had the highest relative incidence of CHE (88%), while those with a household economic status of quintile 1or 4, a household size of 3 or more persons, and a health check-up in the last 12 months had the lowest incidence of CHE (58%).

### Binary logistic regression model for the probability of incurring CHE

Whether CHE occurred was used as the response variable, and nine statistically significant variables for univariate analysis were included as independent variables. The variable screening was performed using the forward stepwise LR method, and the criteria for including and excluding were 0.05 and 0.10, accordingly. Households with larger household sizes (3 or more than 3) were less likely to experience CHE(OR = 0.207). The risk to occur CHE in households with 2, 3 and more than 3 members was 0.790 and 0.207 times higher than in those living alone. There was a significant negative correlation between the incidence of CHE and household economic status. It is obvious that CHE was less likely to occur in wealthier households. Besides, we can also see that patients with 3 or more 3 types of chronic diseases were associated with a high risk of CHE(OR = 3.783). The risk of CHE in patients with 2, 3, or more chronic diseases was 1.807 times and 1.724 times higher than in people with diabetes only (See Table 5).

**Table 5:** Binary logistic regression model for probability of incurring CHE

Variables	β	P	OR	95%CI	
Household size(1)					
2	-0.236	0.608	0.790	0.320	1.950
3 or more	-1.576	0.002	0.207	0.077	0.553
Household economic status^a (^Quintile (1)					
Quintile 2	-1.397	0.003	0.247	0.100	0.613
Quintile 3	-1.436	0.001	0.238	0.104	0.547
Quintile 4	-0.776	0.071	0.460	0.198	1.070
Quintile 5	-1.915	0.000	0.147	0.058	0.373
Number of chronic diseases^b^ (1)					
2	0.592	0.075	1.807	0.943	3.463
3 or more	1.331	0.001	3.783	1.724	8.304

^a^Quintile 1 was the poorest and Quintile 5 was the wealthiest

^b^1 meant patients with only diabetes; 2 meant diabetics suffer from another chronic disease; 3 or more meant diabetic patients with two or more chronic diseases

### ROC analysis

**Fig. 5:**
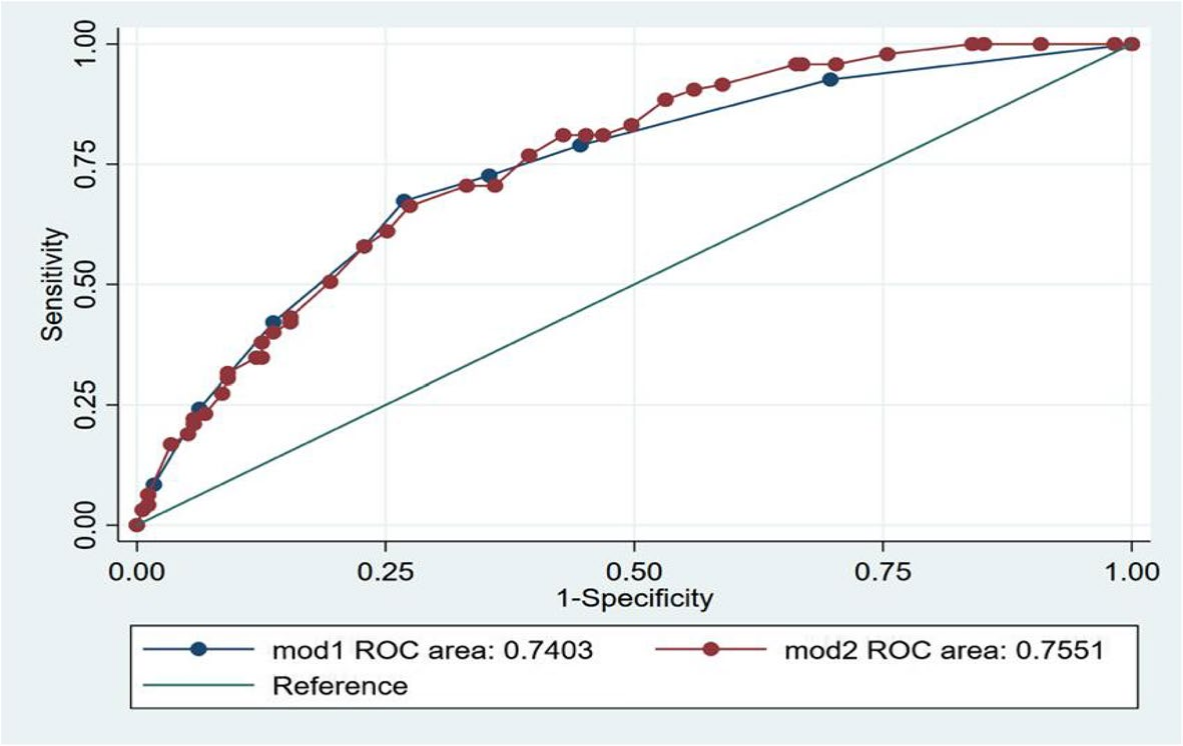
ROC comparison between the two models. Note: Mod1 referred to decision tree, and mod2 referred to logistic regression analysis

AUC was used in this study to determine the superiority of using decision tree model and logistic model to analyze the influencing factors. Usually the closer the AUC is to 1, the higher the accuracy and reliability of the model. The AUC for the decision tree model was 0.7403, and the AUC for the logistic model was 0.7551. The ROC curves were plotted with the predicted probabilities from the decision tree model and the logistic regression model. The ROC curves of both models were relatively close and above the reference line. The two models could be combined in related studies to explore the influential elements collectively (See Fig. 5).

## Discussion

In recent years, the prevalence of diabetes has been increasing, and the number of cases in China is the highest in the world [1]. Diabetes and its complications affect patients' quality of life and lead to a reduction in life expectancy. The economic burden caused by diabetes and the resulting economic risks has received increasing attention from all walks of life. However, most studies tend to be more oriented toward healthy populations, and research on the occurrence and equity of catastrophic health expenditures in specific regions and among patients with specific diseases is limited. Through a timely assessment of the current situation of CHE in diabetic households in the northwest region of China, we identify relevant influencing factors and make policy recommendations that will help improve the health status and quality of life of people with diabetes, the ability of people with diabetes and their families to cope with the economic risk of disease.

### Households with people with diabetes were more likely to incur CHE

Measuring the depth and breadth of CHE, the relevant results were higher than those of other relevant studies in China [23, 24] and in other developing countries [25, 26]. This may be related to the long treatment period, high cost, and complications of chronic diseases, while having a chronic disease may stimulate an increased demand for patients to seek medical services. In the 6th health service survey of Gansu Province, the number of rural respondents was much higher than that of urban respondents. Relevant studies have shown that the prevalence of diabetes in urban areas was higher than that in rural areas, which may lead to the lower measurement results of this study than the actual situation [27]. In addition, CI < 0 indicated that households with people with diabetes at a lower economic level were more likely to experience CHE. And because Gansu Province is an economically less developed region in China, this may be the reason for higher incidence of CHE. Suppose healthcare expenditures account for a high percentage of disposable household income. In contrast, the opportunities for household members to obtain education and generate wealth will be significantly reduced. If this phenomenon continues to develop, it will inevitably affect the quality of life. In summary, CHE is relatively more likely to occur in households with people with diabetes, and this phenomenon was more pronounced in less economically developed areas the high healthcare expenditures may affect the quality of household living standards.

### The low economic level was a risk factor for the occurrence of CHE

In this study, both the decision tree model and the logistic model showed that the worse the economic status of the household, the more likely it was to incur CHE. Many studies have shown that the risk of incurring CHE was associated with household economic status, and the incidence of CHE was lower in households with wealthy economic levels than in poor households [28–30]. This may be because chronic diseases raised the demand for household health services, which led to an increase in healthcare visits and corresponding increases in outpatient costs and hospitalization costs, thus increasing the financial burden of illness on households. A study of 11 Asian countries found that CHE increased the incidence of poverty, with 78 million inhabitants falling into poverty due to excessive health expenditures [31]. Also, the occurrence of CHE was an essential cause of household poverty due to illness [32].

### Household composition status had an impact on the occurrence of CHE

The household composition includes the household size, the disease status of each individual household member, and the presence of the elderly in the household. The household is the first line of defense for individuals against the occurrence of catastrophic health expenditures. This study showed that household size was a protective factor for the occurrence of CHE and that the incidence and average intensity of household risk of CHE was reduced with larger households. This was consistent with previous research findings that the number of household members was higher, the stronger the social support network was, and the greater the household's ability to resist risk [33]. The other study also showed that households with older adults were at greater risk of CHE [23]. There were two main reasons: on the one hand, People who enter the old age stage show a declining trend in all aspects of physiological functions, and at the same time, the probability of suffering from various diseases increases; on the other hand, from the perspective of socio-economic development, entering the old age stage is a significant turning point, most people face retirement and a reduction in work opportunities, which means a reduction in economic income, and the elderly are also chronic diseases The elderly are also a high-risk group for the occurrence of chronic diseases. However, in the study, no correlation has been found between the presence of an older person (> 65 years) in the household and the occurrence of CHE.

### Chronic comorbidity was a risk factor for CHE

Both decision tree and logistic models indicated the occurrence of CHE in households with multiple concurrent chronic diseases. This finding was generally compatible with existing national and international studies: A survey of residents over 50 years of age in Indonesia showed that local residents with three or more chronic diseases had 1.69 folds higher risk of CHE [30]. Suffering from multiple chronic diseases at the same time will generate more and additional health resource consumption compared to the simple sum of chronic diseases alone. Therefore, what we should focus on is the chronic disease co-patient population. By strengthening risk-front awareness and measures, the use of health service resources can be effectively conserved and rationally planned, and it is a scientific guide for targeted risk reduction of the occurrence of CHE.

### The physical examination had an influence on the occurrence of CHE

The decision tree showed that the incidence of CHE of households with a medical examination in the last 12 months was lower than that of households without a medical examination, regardless of the household's economic status and size. Some studies showed that early and appropriate interventions for people with diabetes could significantly minimize the economic risk of the disease [34]. At the same time, through health checks, patients can learn and update their knowledge about diabetes and other chronic diseases promptly. Besides, Medical staff can timely raise patients' health awareness and reasonably intervene in patients' risky behaviors, which can effectively prevent complications or slow down the progression of their occurrence. The comprehensive understanding and awareness of household members about the economic risk of the disease at the present stage can also minimize the prevention and reduction of the severe economic burden caused by the deterioration of the disease and significantly reduce the possibility of the occurrence of CHE.

There are some strengths of this study that are worth noting. Compared to other related studies, the Northwest of China is an economically underdeveloped region with a high prevalence of diabetes and a high economic burden of the disease. Nevertheless, there have been few studies on CHE for people with diabetes. This study focuses on the analysis of incidence of CHE, AGCHE, and RGCHE for a specific chronic disease in less economically developed regions of China. Second, this study uses a Pareto chart to analyze the main intervals in which CHE occurs, providing evidence to protect low- and middle-income households from falling into economic poverty. Finally, this study analyzed the influencing factors through decision trees and logistic models to provide clues for reducing the risk of CHE. However, this study also has some limitations. Following a sample survey under strict procedures; 270 households were finally selected for the study, a slightly inadequate sample size. Besides, the data used in this study lacked a timeline, so the findings were interpreted as associations rather than causal impacts. The following ideas will be expanded to explore the economic burden of disease and the associated economic risk of disease under different timelines.

## Conclusions

Compared with other diseases, treating chronic diseases is often a lifelong and continuous process, bringing a vast amount of medical expenses. At the same time, it has also become a key factor hindering the improvement of household living standards. There is a lack of adequate financial health protection for a household with people with diabetes in the northwest area of China, even though there is a high coverage rate with medical insurance. In summary, our study found that incidence of CHE, AGCHE, and RGCHE were higher than the results of other similar studies under the same criteria. The Pareto chart showed that the annual per capita household income corresponding to the A interval was even, suggesting that the focus should be on low- and middle-income diabetic households. Both decision tree and logistic models revealed that economic level remained the main factor contributing to the occurrence of CHE in households with diabetes. At the same time, it was also found that households with small household members with diabetic comorbidities are more likely to incur CHE. The decision tree model also suggested that among the influencing factors affecting the occurrence of CHE, there were interactions between household economic status, household size, whether health checks were performed in the last 12 months, and the number of chronic disease types. It is necessary to promote the need for timely assessment of the economic risk of disease, to improve the economic situation of patients' households, reduce the likelihood of CHE in households with people with diabetes, and to improve the ability to cope with the economic risk of disease.

## Data Availability

The datasets used and/or analysed during the current study are available from the corresponding author on reasonable request.

## Abbreviations

CND: Chronic Non-communicable Disease
CHE: Catastrophic Health Expenditure
AGCHE: Average Gap of Catastrophic Health Expenditure
RGCHE: Relatively Gap of Catastrophic Health Expenditure
CI: Concentration Index
IDF: International Diabetes Federation
NHSS: National Health and Services Survey
AUC: Area Under Curve
